# The landscape of tumors-infiltrate immune cells in papillary thyroid carcinoma and its prognostic value

**DOI:** 10.7717/peerj.11494

**Published:** 2021-05-21

**Authors:** Yanyi Huang, Tao Yi, Yushu Liu, Mengyun Yan, Xinli Peng, Yunxia Lv

**Affiliations:** 1 Department of Thyroid Surgery, Second Affiliated Hospital of Nanchang University, Nanchang, Jiangxi, China; 2 Nanchang University, The Second Clinical Medicine College, Nanchang, Jiangxi, China; 3 Department of Otolaryngology, People’s Hospital of Yichun, Yichun, Jiangxi, China; 4 Nanchang University, The First Clinical Medicine College, Nanchang, Jiangxi, China; 5 Department of Otolaryngology, First Affiliated Hospital of Nanchang University, Nanchang, Jiangxi, China

**Keywords:** Papillary thyroid carcinoma, Tumors-infiltrate immune cells, Prognostic model

## Abstract

**Introduction:**

Thyroid cancer is a very common malignant tumor in the endocrine system, while the incidence of papillary thyroid carcinoma (PTC) throughout the world also shows a trend of increase year by year. In this study, we constructed two models: ICIscore and Riskscore. Combined with these two models, we can make more accurate and reasonable inferences about the prognosis of PTC patients.

**Methods:**

We selected 481 PTC samples from TCGA and 147 PTC samples from GEO (49 samples in GSE33630, 65 samples in GSE35570 and 33 samples in GSE60542). We performed consistent clustering for them and divided them into three subgroups and screened differentially expressed genes from these three subgroups. Then we divided the differential genes into three subtypes. We also distinguished the up-regulated and down-regulated genes and calculated ICIscore for each PTC sample. ICIscore consists of two parts: (1) the PCAu was calculated from up-regulated genes. (2) the PCAd was calculated from down-regulated genes. The PCAu and PCAd of each sample were the first principal component of the relevant gene. What’s more, we divided the patients into two groups and constructed mRNA prognostic signatures. Additionally we also verified the independent prognostic value of the signature.

**Results:**

Though ICIscore, we were able to observe the relationship between immune infiltration and prognosis. The result suggests that the activation of the immune system may have both positive and negative consequences. Though Riskscore, we could make more accurate predictions about the prognosis of patients with PTC. Meanwhile, we also generated and validated the ICIscore group and Riskscore group respectively.

**Conclusion:**

All the research results show that by combining the two models constructed, ICIscore and Riskscore, we can make a more accurate and reasonable inference about the prognosis of patients with clinical PTC patients. This suggests that we can provide more effective and reasonable treatment plan for clinical PTC patients.

## Introduction

Thyroid cancer is the most common malignancy of the endocrine system (Cabanillas, McFadden & Durante, 2016), and the global incidence of papillary thyroid carcinoma (PTC) has continued to increase annually, making it a hotspot for research on tumors of the endocrine system. PTC accounts for more than 70% of thyroid malignancies (Lloyd, Buehler & Khanafshar, 2011). Generally, PTC patients have a good survival prognosis, with less than a 2% mortality rate after 5 years (Abdullah et al., 2019). Therefore, more detailed classifications of PTC patients and targeted treatment can improve the quality of life for these patients.

The immune system plays an important role in the occurrence and development of tumors (Scouten & Francis, 2006). On one hand, the immune system prevents the occurrence and development of tumors by monitoring and removing tumor cells. On the other hand, the immune system’s ability to clear tumor cells decreases due to various factors and as a tumor develops. Therefore, tumor-infiltrating immune cells are closely associated with tumor development. In particular, the interaction between immune cells and cancer cells contributes to the occurrence and development of cancer (Crespo et al., 2013). The immune system also plays a significant role in potentially screening, controlling, and treating tumors. Additionally, immunotherapy that targets CTLA-4 and PD-L1 checkpoint blocking antibodies (Varricchi et al., 2019) can improve the prognoses of patients with multiple malignancies. Therefore, immunotherapy is considered an effective tumor treatment method with good synergistic survival benefits across a variety of cancers (Antonelli, Ferrari & Fallahi, 2018). However, due to the limitations of this treatment, it is necessary to investigate novel therapeutic markers that could determine PTC subgroups for immunotherapy treatment.

Previous studies have classified the immune subtypes of thyroid cancer (Xie et al., 2020; Zhi et al., 2020) and grouped risk scores (Zhuang et al., 2020), but no comprehensive analysis or comparison of the two approaches have been conducted. This study is the first to focus on these approaches and use them to construct two signatures: ICIscore and Riskscore. ICIscore is the immune signature with lymphocyte infiltration estimation ability while Riskscore is the prognosis signature with disease-free survival (DFS) prediction. Using these two models, we can make more accurate and reasonable inferences about the prognoses of PTC patients. In this study, we also explored the correlation between tumor immune cell infiltration and DFS in tumor patients. Immune systems can show favorable or adverse outcomes, which are exhibited in the form of pro-tumor or anti-tumor activity (Chen et al., 2019; Chen & Mellman, 2017). The results of this study provide a new way to analyze the DFS of tumor patients.

## Materials and Methods

### Data sources and processing

We downloaded clinical and RNA sequencing (RNA-seq) data (Fragments Per Kilobase Million (FPKM) values) about PTC from the Cancer Genome Atlas (TCGA) database (https://portal.gdc.cancer.gov/). From these data, we selected 481 patients with an effective DFS (Kanazawa & Kammori, 2019) for our study. DFS refers to the time from randomization when the disease recurred or when the patient died as a result of disease progression. The microarray datasets (GSE35570, GSE33630, and GSE60542) were downloaded from the Gene Expression Omnibus (GEO) database (https://www.ncbi.nlm.nih.gov/geo/). We used the “ComBat” algorithm (Mansbach et al., 2020) to reduce the batch effects from non-biological technical biases between GSE35570, GSE33630, and GSE60542. We combined these three datasets and referred to them as the GEO combined sets.

### Consistent clustering of tumor-infiltrating immune cells

Using previous pan-cancer analyses (Charoentong et al., 2017), we obtained a gene set of 28 tumor-infiltrating immune cells. We used a Single Sample Gene Set Enrichment Analysis (ssGSEA) algorithm (Yi et al., 2020) and the ‘GSVA’ package (Hänzelmann, Castelo & Guinney, 2013) in R to obtain the enrichment score of each patient. Each sample’s immune and stromal levels (immune score, stromal score, ESTIMATE score, and tumor purity) were obtained using an estimate algorithm (Ke et al., 2020). We performed 50 iterations of consistent clustering with a resample rate of 80% for each patient based on the immune cell infiltration (ICI) pattern using “ConsensusClusterPlus” package (Wilkerson & Hayes, 2010). We used the “pheatmap” package to visualize the clustering results. We designated the three clusters, ICIcluster 1, ICIcluster 2, and ICIcluster 3, according to their ICI levels (from low to high).

### ICI cluster differentially expressed gene (DEG) analysis

Considering that some of the samples had a gene expression level of 0 which would lead to bias in the results, we only selected genes with mean gene expression levels greater than or equal to 0.1 for use in the subsequent study. DEGs were screened and identified from the three ICI clusters using |Log2 fold change (LogFC) > 1.8| and a false discovery rate (FDR) < 0.01,which was implemented by the Wilcox test. Univariate COX analysis (Tibshirani, 2009) was used to determine the prognostic DEGs with *p* < 0.01.

### Identifying gene clustering and ICIscore generation

Using the consistent clustering method, we identified three gene clusters. The genes that positively and negatively correlated with ICIcluster were designated as the upregulated and downregulated genes, respectively. We used a principal-component analysis (PCA) algorithm (Ringnér, 2008; David & Jacobs, 2014; Konishi et al., 2019) to extract the first principal components of the upregulated and downregulated genes. Next, we defined the ICIscore of each patient:

$$ICIscore=\sum PCA_{u}-\sum PCA_{d}$$

where u refers to the upregulated genes and d refers to the downregulated genes. We selected the best cutoff using the “survminer” package. According to the cutoff, we divided the patients into two groups (ICIscore low and ICIscore high).

### Constructing the prognostic signature and Riskscore generation

We randomly divided TCGA patients into two cohorts. Cox proportional hazard models are the most widely-used approach for modeling time to event data. They use the principle of recursive elimination to compute the instantaneous rate of an event occurrence (Asano, Hirakawa & Hamada, 2014). We constructed a 5-mRNA prognostic signature using the COX-PH algorithm. Next, we defined the Riskscore (Lee, 2008) of each patient:

$$Riskscore=\overset{n}{\underset{i=1}{\sum }}Expi\;\times \;Coef$$

where n refers to the number of genes in the signature, Expi refers to the level of gene expression in the signature, and Coef refers to the estimated regression coefficient value from the COX-PH algorithm. According to their Riskscore median, the patients were divided into two groups (Riskscore low and Riskscore high). The signature was also fitted in the testing set and the total set.

### Statistical analysis

All statistical analyses were conducted using R (3.6.1) software. We used the Mann–Whitney *U* test to compare two groups, and the Kruskal–Wallis test to compare more than two groups. The Kaplan-Meier (K-M) survival curves (You et al., 2018) were used to generate different groups of DFS curves. The receiver operating characteristic curves (ROC) (Blangero et al., 2020) was used to identify the Riskscore prognosis. Two-tailed *P* values less than 0.05 were considered statistically significant.

## Results

### The landscape of tumor-infiltrating immune cells

**Figure 1:**
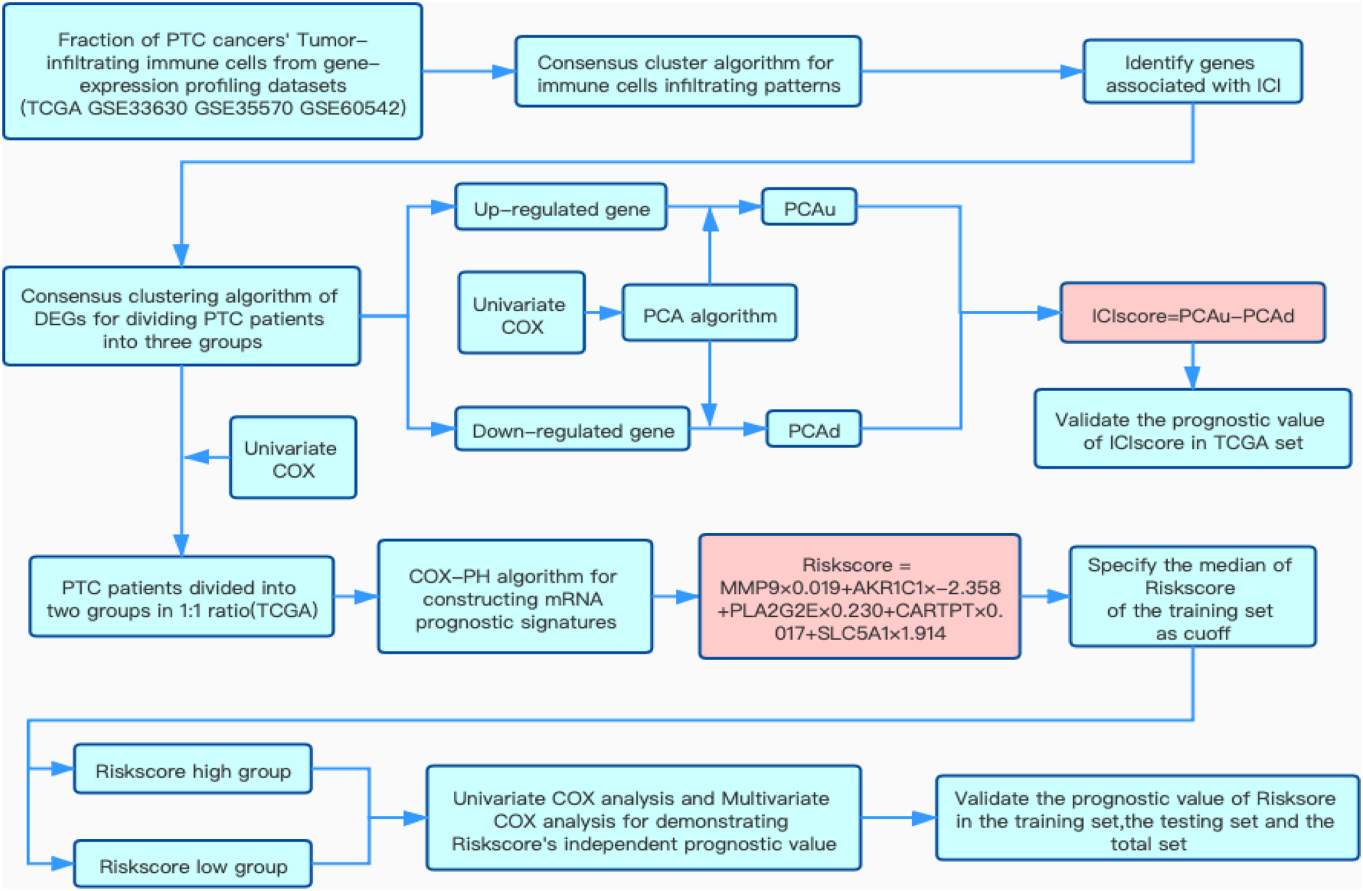
Flow chart of this study. Overview of research design

**Figure 2:**
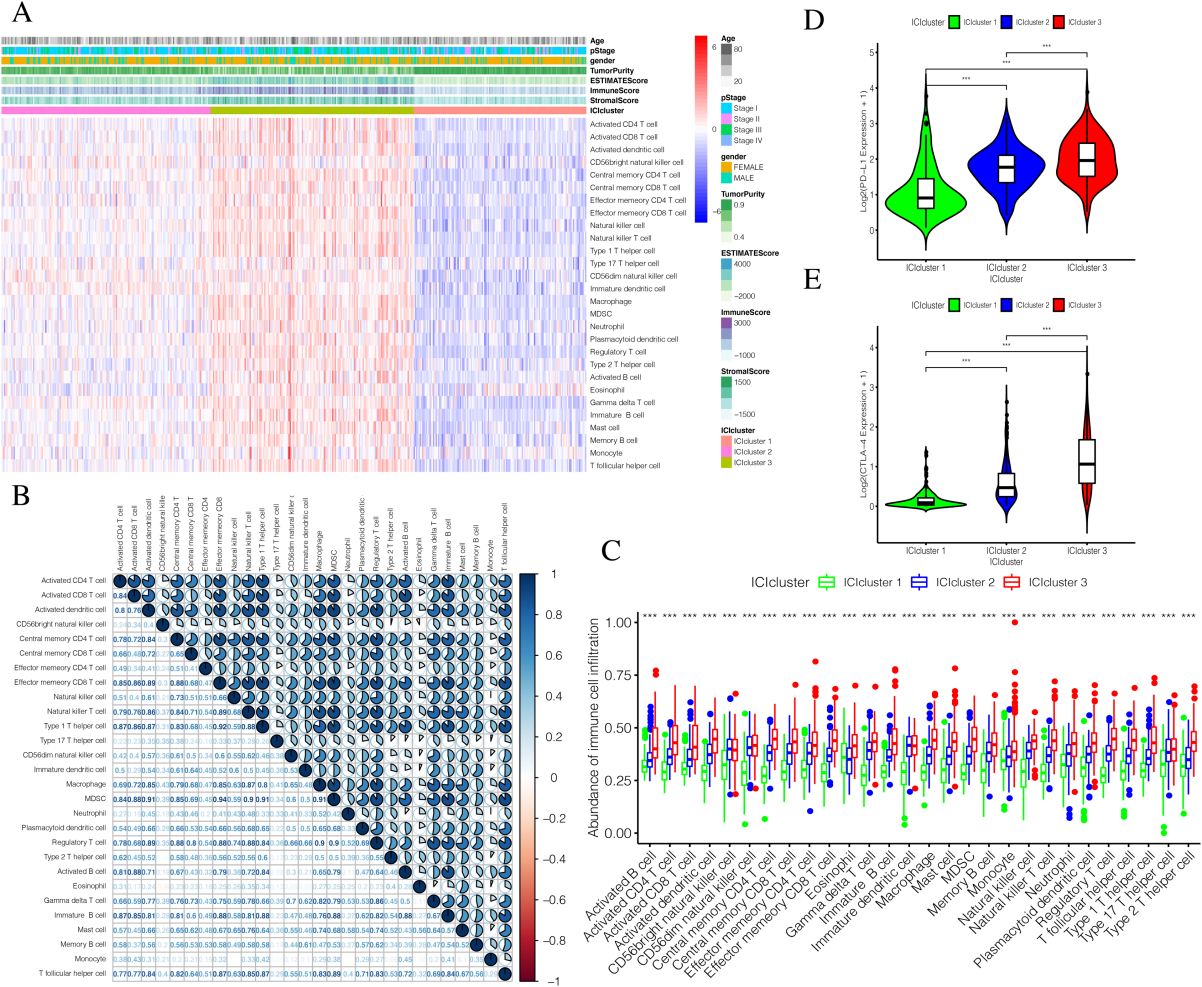
Exploration and validation the correlation between differentiation of ICIclusters and immune cell infiltration. Through ssGSEA, 28 immune-infiltrating cells were enriched. (A) The heat map is included the age, pStage, gender, tumor purity, estimate score, immune score, stromal score and ICIclusters. (B) The correlation matrice of tumor-infiltrating immune cells. (C) The comparation of enrichment score among three ICIclusters. (D) The expression level of PD-L1 in each combination was compared. (E) The expression level of CTLA4 in each combination was compared.

**Figure 3:**
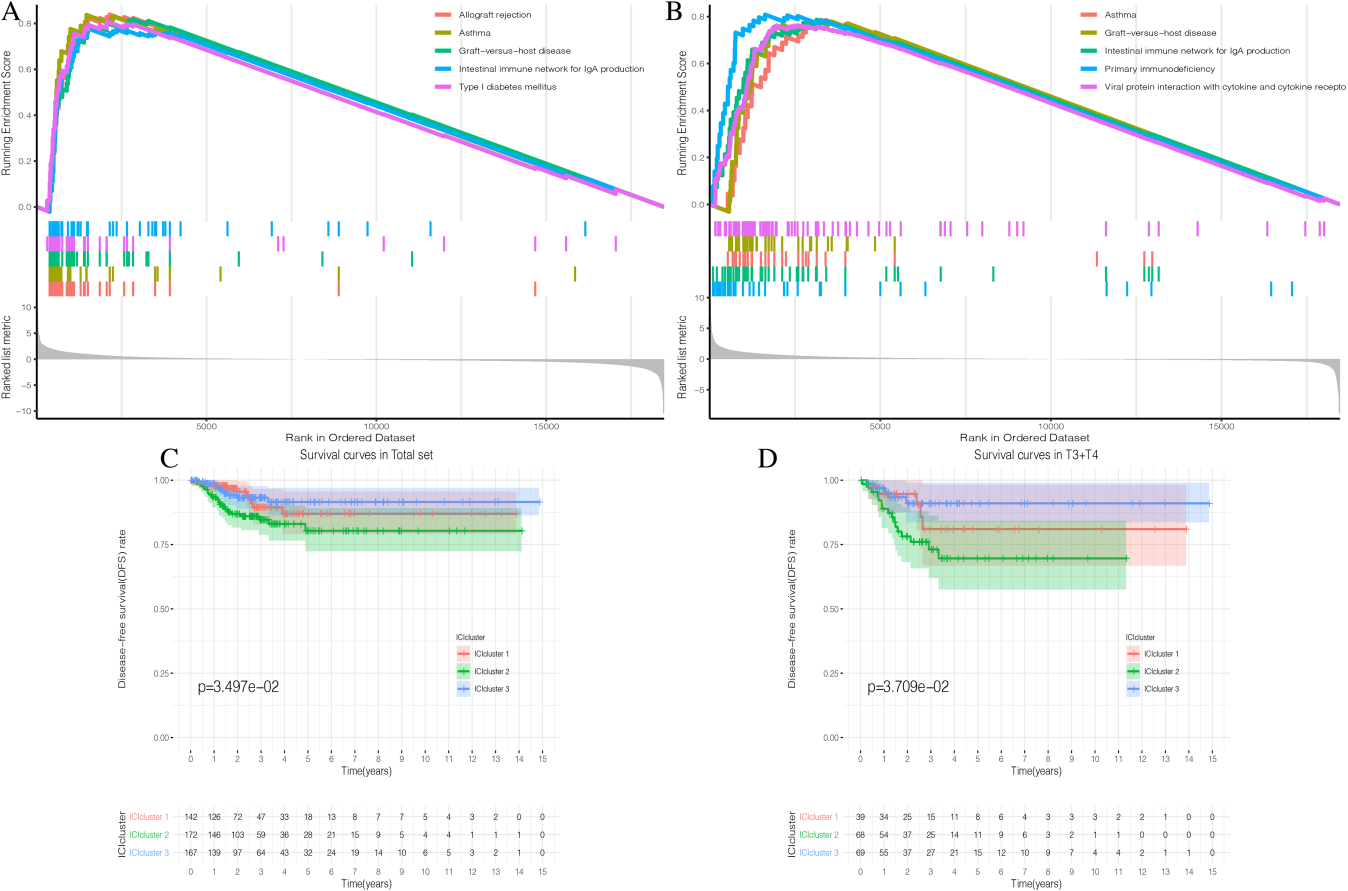
Prognostic correlation analysis of three ICIclusters. (A and B) Enrichment plots showing different enrichment of different diseases and pathways in the Rank in Ordered Dataset. (C) The Kaplan-Meier (K-M) curves of Disease-free survival (DFS) in Total set (D) The Kaplan-Meier (K-M) curves of Disease-free survival (DFS) in T3 + T4

The detailed flow chart of this study is shown in Fig. 1. Using the gene set associated with 28 types of infiltrating immune cells (Table S1) (Charoentong et al., 2017) and the ssGSEA algorithm, we obtained the enrichment score of the tumor-infiltrating immune cells in each PTC sample. Using consistent clustering, we divided the PTC patients into three ICI subtypes (Fig. 2A and Fig. S1). Based on the correlation analysis, we generated a correlation matrix of the tumor-infiltrating immune cells (Fig. 2B). Almost all of the items showed a positive correlation. The most significant correlation was shown between Myeloid-derived suppressor cells (MDSC) and other cells, especially MDSCs and effector memory CD8 T cells (Khaled, Ammori & Elkord, 2013; Lechner et al., 2011). WE used the Kruskal–Wallis test to compare the enrichment score across the three ICI subtypes (Fig. 2C) and we found that there were significant differences across the three ICI groups, indicating that our clustering was successful. Additionally, we performed pairwise combinations of the three ICI subtypes, and the Mann–Whitney *U* test to compare the expression levels of two immune checkpoint-related genes (PD-L1 and CTLA-4) in every combination (Figs. 2D and 2E). To explore the biological processes of the different ICIclusters, we used GSEA enrichment analysis (Subramanian et al., 2005) on the different ICIclusters. The ICIcluster 2 pathway was significantly enriched compared to ICIcluster 1 (Fig. 3A). Similarly, Fig. 3B shows that the ICIcluster 3 pathway was significantly enriched compared to ICIcluster 2. Detailed enrichment results can be seen in Table S2.

The same approach was applied to the GEO combined sets. We used consistency clustering to divide patients into three groups (Fig. S2A). The correlation analysis of the 28 types of tumor-infiltrating immune cells is shown in Fig. S2B. Significant differences were shown across the three ICIclusters (Fig. S2C), which was consistent with the performance of TCGA set. In the analysis of the immune checkpoint-related genes (PD-L1 and CTLA-4), there were also significant differences across the ICIclusters (Figs. S2D–S2F). GSEA enrichment analysis was also performed for the different ICIclusters, and the results can be found in Figs. S3A and S3B. Detailed enrichment results can be seen in Table S3. In the most significant Top 10 enrichment pathway, the GEO combined set and TCGA set were consistent with one another. This suggests that our ICIclusters represented the tumor-infiltrating immune cell landscape of PTC patients and will lay the foundation for subsequent analysis.

### Stratified DFS analysis of the three ICI subtypes

The three ICI subtypes showed different DFS conditions compared to the total set (Fig. 3C). ICIcluster 3 had the most significant immune cell infiltration and was associated with the best prognosis. It is notable that ICIcluster 2 showed moderate immune cell infiltration but was associated with the worst prognosis. Although ICIcluster 1 had the lowest immune cell infiltration, it was associated with a moderate prognosis. Such results were also reflected in the Age <45 and T3 + T4 groups (Fig. 3D). This indicates that high or low immune function has the same influence on tumor prognosis, and will ultimately inhibit tumor development.

### Identifying DEGs associated with the three ICI subtypes

**Figure 4:**
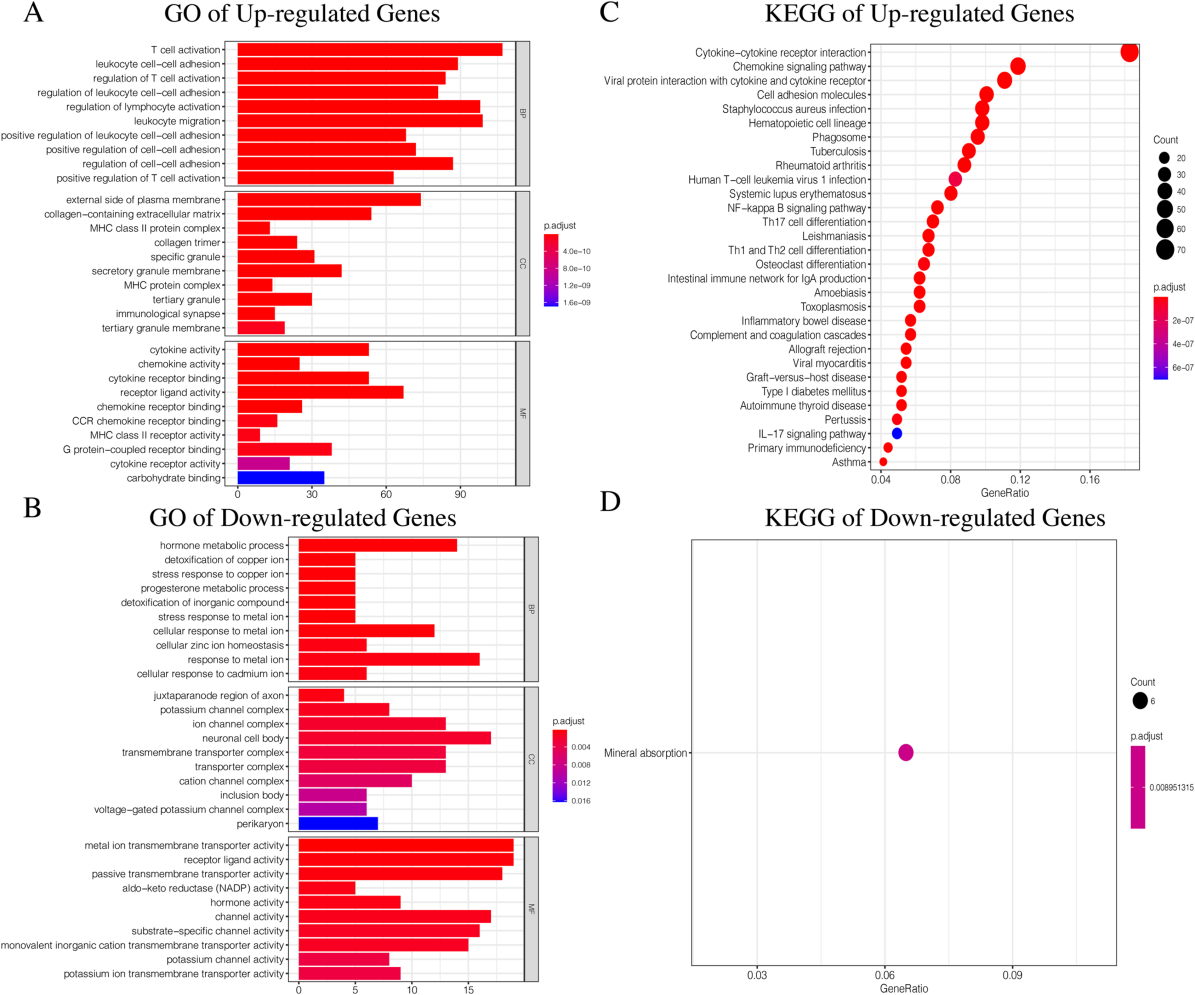
The GO and KEGG enrichment analysis of Up-regulated Gene and Down-regulated Gene. (A) The GO enrichment analysis of Up-regulated Gene. (B) The GO enrichment analysis of Down-regulated Gene. (C) The KEGG enrichment analysis of Up-regulated Gene. (D) The KEGG enrichment analysis of Down-regulated Gene.

Since TCGA set had complete clinical information and prognostic indicators, we conducted subsequent studies using this dataset. In order to make the results more accurate, we selected genes with average gene expression levels greater than or equal to 0.1 in order to determine the underlying molecular differences across the different ICI subtypes. We used the Wilcox-test to identify the DEGs associated with the three ICI subtypes. With |LogFC > 1.8| and FDR < 0.01, we obtained 982 DEGs: 752 upregulated genes and 230 downregulated genes (Fig. S4, Table S4). Upregulated genes were those that were highly expressed in at least two ICI subtypes with higher immune cell infiltration. The rest were labeled downregulated genes. To determine the underlying biological functions of the DEGs, we conducted Gene Ontology (GO) and Kyoto Encyclopedia of Genes and Genomes (KEGG) enrichment analyses (Xing et al., 2020; Wang et al., 2020) of the upregulated genes and downregulated genes, respectively. The most significant enrichment results are summarized in Figs. 4A–4D, and detailed content is provided in Table S5. Using *P* < 0.01 as the cutoff, we performed univariate COX analysis to identify the DEGs associated with prognosis. Finally, we obtained 15 DEGs: 10 upregulated and five downregulated genes (Table 1).

**Table 1:** Univariate COX analysis of differentially expressed gene.

Symbol	Entrez ID	HR	95% CI		*P* value	Gene type
			Low	High		
AKR1C1	1645	0.068	0.010	0.437	0.005	Down-regulated Gene
MYH3	4621	1.109	1.060	1.159	<0.001	Down-regulated Gene
CSMD1	64478	1.239	1.117	1.374	<0.001	Down-regulated Gene
CARTPT	9607	1.003	1.001	1.005	0.003	Down-regulated Gene
SLC5A1	6523	1.222	1.069	1.396	0.003	Down-regulated Gene
MMP9	4318	1.012	1.003	1.021	0.010	Up-regulated Gene
FN1	2335	1.000	1.000	1.001	<0.001	Up-regulated Gene
PLA2G2E	30814	1.231	1.107	1.369	<0.001	Up-regulated Gene
C2CD4A	145741	1.108	1.051	1.169	<0.001	Up-regulated Gene
DAW1	164781	3.126	1.322	7.395	<0.001	Up-regulated Gene
MUC21	394263	1.026	1.009	1.042	0.002	Up-regulated Gene
AHNAK2	113146	1.054	1.017	1.093	0.004	Up-regulated Gene
IL1RN	3557	1.062	1.024	1.101	0.001	Up-regulated Gene
DCSTAMP	81501	1.007	1.002	1.013	0.005	Up-regulated Gene
SLC34A2	10568	1.001	1.000	1.001	0.008	Up-regulated Gene

**Note:**

HR, hazard ratio; CI, confidence interval.

### Identifying immune gene subtypes

**Figure 5:**
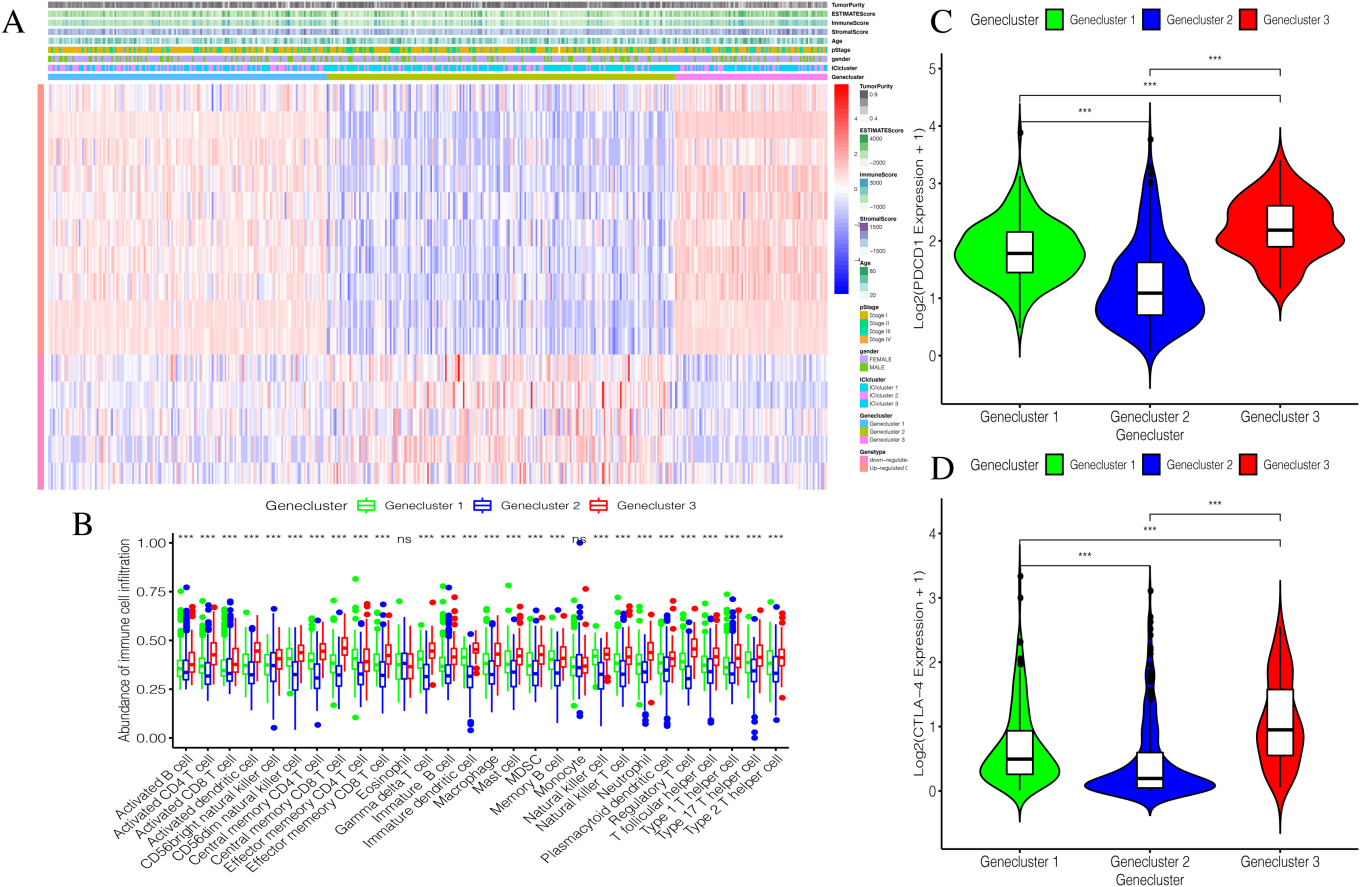
Establishment and verification of Geneclusters. (A) The heat map is included the tumor purity, estimate score, immune score, stromal score, age, pStage, gender, ICIcluster and Genecluster. (B) Comparison of infiltration levels of immune cells in three Geneclusters. (C) The expression level of immune checkpoint related gene PD-L1. (D) The expression level of immune checkpoint related gene CTLA4.

**Figure 6:**
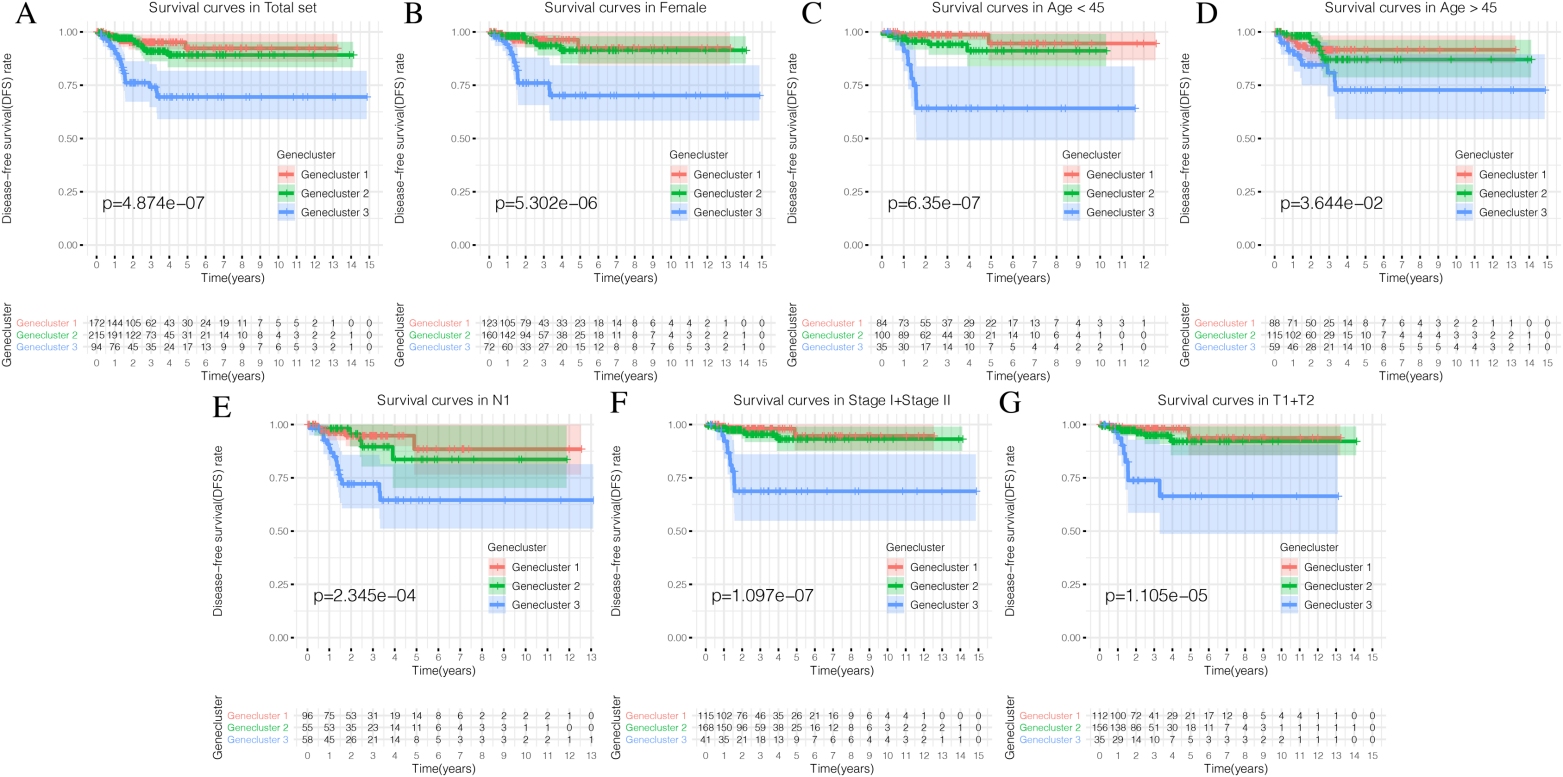
Prognostic differences of the three Geneclusters. (A) The Kaplan-Meier (K-M) curves of Disease-free survival (DFS) in total set. (B) The Kaplan-Meier (K-M) curves of Disease-free survival (DFS) in female. (C) The Kaplan-Meier (K-M) curves of Disease-free survival (DFS) in age <45.(D) The Kaplan-Meier (K-M) curves of Disease-free survival (DFS) in age >45. (E) The Kaplan-Meier (K-M) curves of Disease-free survival (DFS) in N1. (F) The Kaplan-Meier (K-M) curves of Disease-free survival (DFS) in Stage1 + Stage2. (G) The Kaplan-Meier (K-M) curves of Disease-free survival (DFS) in T1 + T2.

Using our consistent cluster analysis of the 15 genes listed above, we obtained three gene clusters (Fig. 5A). In order to explore the abundance of immune cell infiltration across the three gene subtypes, we performed Kruskal–Wallis tests on the three subtypes. We found that Genecluster 1 and Genecluster 3 had more tumor-infiltrating immune cells and that Genecluster 3 was more significant than Genecluster 1 (Fig. 5B). This difference at the immune level was also shown in the expression levels of the two immune checkpoint-related genes (PD-L1 and CTLA-4; Figs. 5C–5D). Furthermore, we also explored the prognostic differences across the three gene subtypes. Genecluster 1 and 2 were associated with a favorable prognosis while Genecluster 3 was associated with the worst prognosis (Fig. 6A). It is worth noting that in addition to the worst prognosis, Genecluster 3 also had the most immune infiltrating cells. The immune system’s specific mechanism involved with tumor occurrence and development is worthy of further investigation, as its activation may have both favorable and unfavorable results. According to clinical stratification studies, similar results can also be seen in patients who were female, age <45, age >45, N1, Stage I + Stage II, and T1 + T2 (Figs. 6B–6G).

### Generation and validation of the ICIscore groups

**Figure 7:**
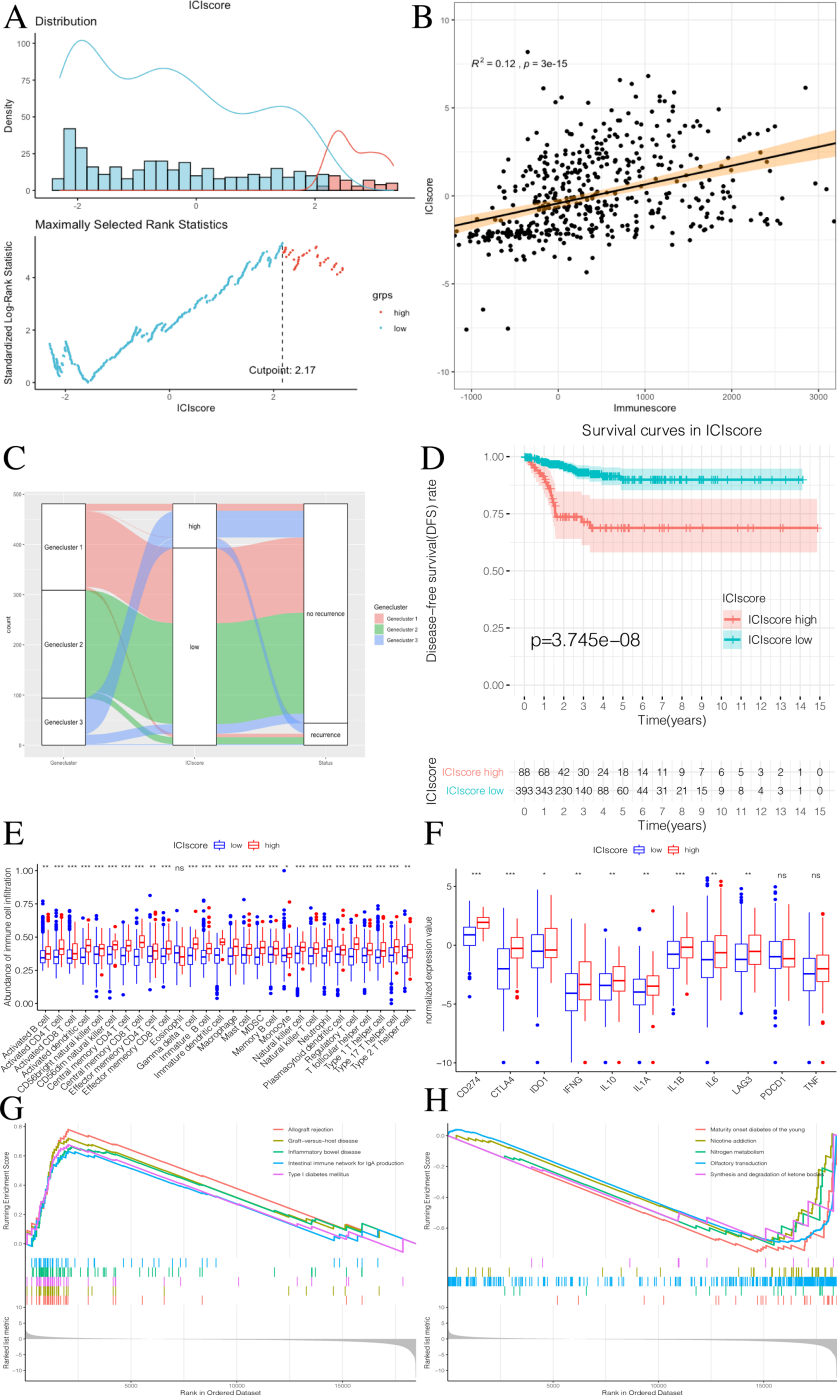
Construction of ICIscore and its clinical prognostic analysis. (A) The construction of ICIscore with high and low grouping model. (B) The scatter plot showing the relationship between ICIscore and Immunesocre. (C) Alluvial diagram of Genecluster distribution in groups with different Geneclusters, ICIscores, and survival outcomes. (D) The Kaplan-Meier (K–M) survival curves in ICIscore high and low grouping. (E) Differences in immune cell infiltration expressed in high and low ICIscore subgroups. (F) Immune-checkpoint-relevant genes and immune-activation-relevant genes expressed in high and low ICIscore subgroups. (G and H) Enrichment plots showing different enrichment of different pathways in the ICIscore high and low grouping.

We used the PCA algorithm to calculate the ICIscore for each PTC sample. The ICIscore consisted of two parts: (1) the PCAu calculated from upregulated genes and (2) the PCAd calculated from downregulated genes. The PCAu and PCAd of each sample were the first principal components of the relevant gene. Finally, using the ICIscore formula, we obtained the ICIscore of each sample. With the “survminer” package, we calculated the optimal ICIscore cutoff as 2.17 (Fig. 7A) and divided the patients into two groups (ICIscore high and ICIscore low). Since the ICIscore correlated with immune status and the immune score reflected the level of immune cell infiltration, it was reasonable to believe that there was a correlation between these values. In order to explore this relationship, we drew a scatter plot (Fig. 7B) that showed a highly positive correlation between the two, indicating that ICIscores could reflect the level of immune cell infiltration to some extent. Although immune scores do not have prognostic value, we found that ICIscores did correlate with prognosis in a follow-up study. The distribution of all patients is shown in Fig. 7C. Patients with high ICIscores were mainly distributed in Genecluster 3 with poor prognosis predicted. To determine whether different ICIscore groups had different prognoses, we plotted the K-M survival curve. We found that the ICIscore high group showed significantly poorer prognoses (Fig. 7D). To evaluate whether ICIscores reflected differences in immune levels across the groups, we compared the tumor-infiltrating immune cells between the two groups using a Mann–Whitney *U* test (Fig. 7E). Additionally, we selected PDCD1, CD274, CTLA-4, LAG3, and IDO1 as the immune checkpoint-related genes and IL1A, IL1B, IL6, IL8, IFNG, and TNF as the immune active factors (Ayers et al., 2017; Deng et al., 2012). The Mann–Whitney *U* test showed that most immune checkpoint-related genes, except PDCD1 and TNF, and immune active factors were highly expressed in the ICIscore high group (Fig. 7F). Based on the GSEA results, we also found that the “allograft rejection” and “graft-versus-host disease” pathways were significantly enriched in the ICIscore high group, while the “maturity onset diabetes of the young” and “olfactory transduction” pathways were significantly enriched in the ICIscore low group (Figs. 7G–7H, Table S6).

### Generation and validation of the Riskscore group

**Figure 8:**
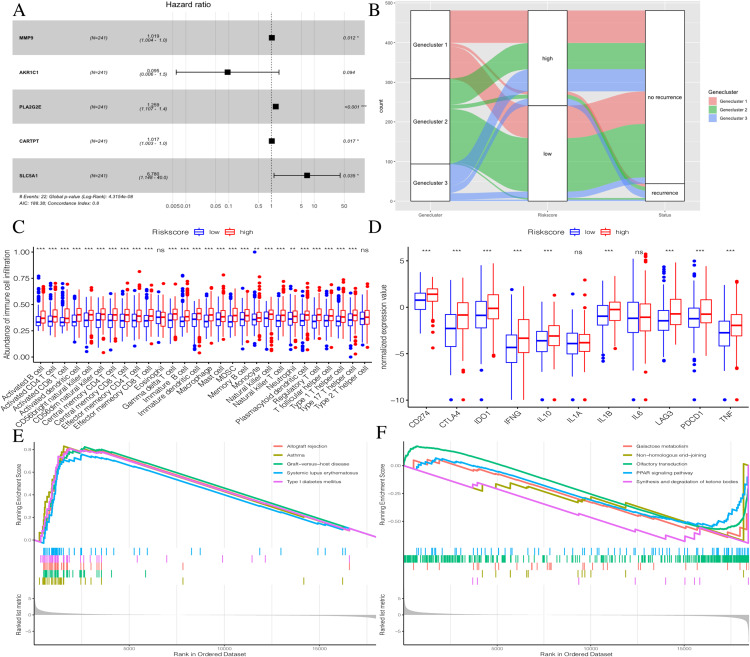
Construction of Riskscore. (A) The construction of a 5-mRNA prognostic signature. (B) Alluvial diagram of Genecluster distribution in groups with different gene clusters, risk, and Disease-free survival (DFS) outcomes. (C) Differences in immune cell infiltration expressed in high and low Riskscore.(D) Immune-checkpoint-relevant genes and immune-activation-relevant genes expressed in high and low Riskscore. (E and F) Enrichment plots showing different enrichment of different diseases in the Riskscore high and Riskscore low.

To further explore the potential prognostic value of these genes, we randomly divided TCGA PTC patients into two groups at a 1:1 ratio. We used the COX-PH algorithm to construct a 5-mRNA prognostic signature (Fig. 8A, Table 2). We specified the Riskscore median cutoff of the training set to fit the testing set and the total set. Riskscore = MMP9 × 0.019 + AKR1C1 × −2.358 + PLA2G2E × 0.230 + CARTPT × 0.017 + SLC5A1 × 1.914. Previous literature suggested that these five genes were closely related to cancer prognosis. In addition, it has been reported that the elevated MMP-9 level in PTC patients is defined as a malignant factor of PTC (Zarkesh et al., 2018) and may be involved in the mechanism of ROCK1 in the occurrence and development of PTC (Luo et al., 2017). AKR1C1, PLA2G2E, CARTPT, and SLC5A1 have been shown to be abnormally expressed in cancer and influence prognosis (Zeng et al., 2017; Sato et al., 2014; Burgos, Iresjö & Smedh, 2016; Gao et al., 2019; Mojica, Luna-Vital & Gonzalez de Mejia, 2018; Lei et al., 2016).

**Table 2:** COX-PH algorithm constructed 5-mRNA signature.

Symbol	Coef	HR	95% CI		*P* value
			Low	High	
MMP9	0.019	1.019	1.004	1.034	0.012
AKR1C1	−2.358	0.095	0.006	1.497	0.094
PLA2G2E	0.230	1.259	1.107	1.431	<0.001
CARTPT	0.017	1.017	1.003	1.032	0.017
SLC5A1	1.914	6.780	1.148	40.034	0.035

**Note:**

Coef, Coefficient of the model; HR, hazard ratio; CI, confidence interval.

The distribution of all patients is shown in Fig. 8B. Patients with high Riskscores had poor prognosis. Before verifying the signature’s prognostic effect, we compared the immune cell infiltration between the two Riskscore groups (Fig. 8C). There was a significant difference in the level of immune cell infiltration between the two groups, and the Riskscore high group showed more immune cell infiltration. Additionally, most immune checkpoint-related genes and immune active factors, except IL1A and IL6, were highly expressed in the Riskscore high group (Fig. 8D). We also performed GSEA enrichment analysis on the two groups. The “allograft rejection” and “asthma” pathways were significantly enriched in the Riskscore high group, while the “synthesis and degradation of ketone bodies” and “ on-homologous and end-joining” pathways were significantly enriched in the Riskscore low group (Figs. 8E and 8F). More details are shown in Table S7.

**Figure 9:**
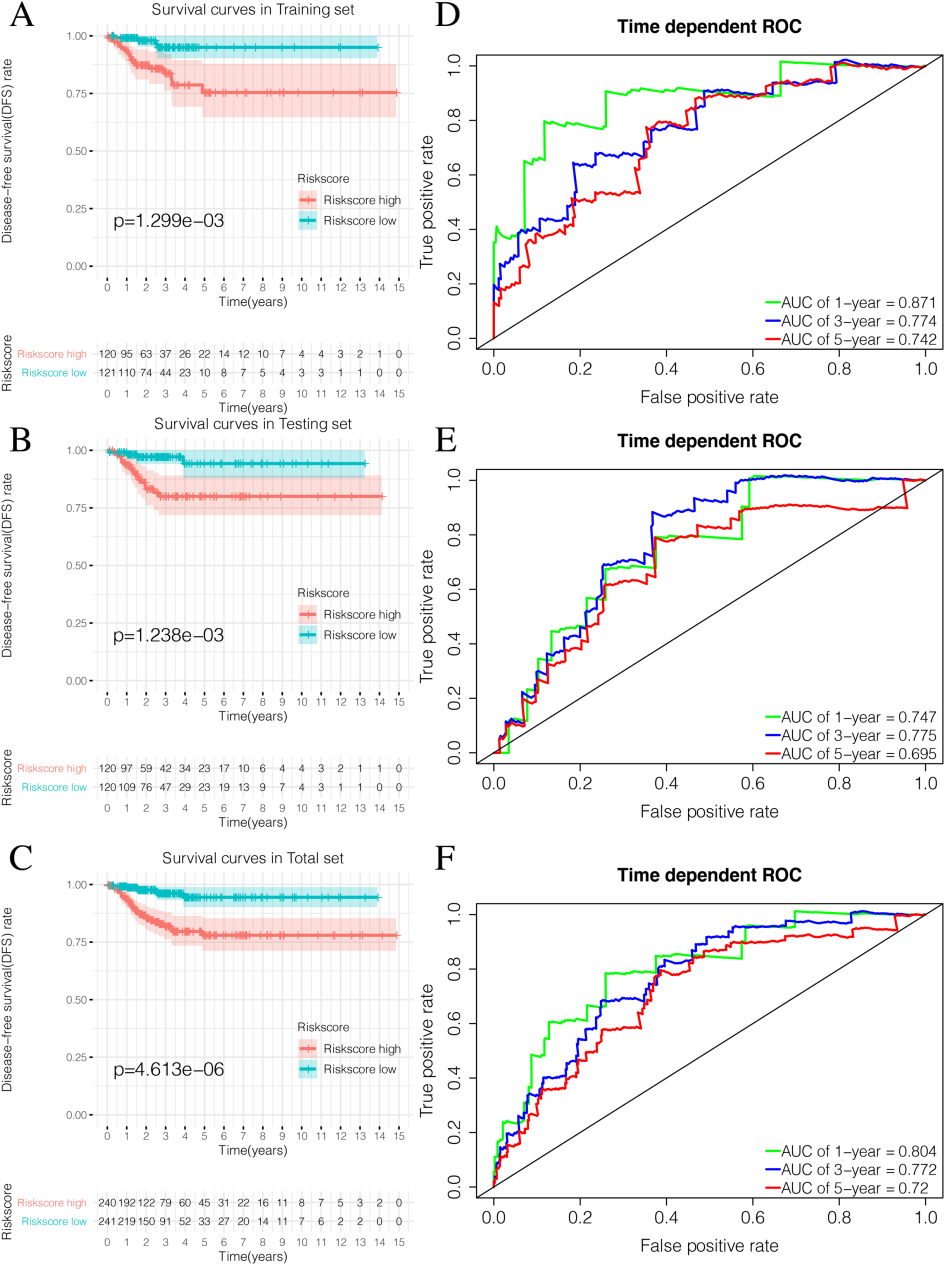
Validation of the prognostic signature of Riskscore. (A) The Kaplan-Meier (K-M) curves of Disease-free survival (DFS) in the training set. (B) The Kaplan-Meier (K-M) curves of Disease-free survival (DFS) in the testing set. (C) The Kaplan-Meier (K-M) curves of Disease-free survival (DFS) in the total set. (D–F) Time-dependent ROC curves in the training set, testing set and the total set at 1-year, 3-year and 5-year.

By plotting K-M survival curves for the training, testing, and the total sets, we found that the Riskscore high group had a significantly poor prognosis (Figs. 9A–9C). In order to reveal the prognostic value of the signature, we verified the prognostic Riskscore value by drawing ROC curves for the different time periods (1-year, 3-years, and 5-years; Figs. 9D–9F). The AUC of the training, testing, and total set were mostly greater than 0.7, indicating that our signature had good prognostic value.

### ICIscore and Riskscore clinical correlation analysis

**Figure 10:**
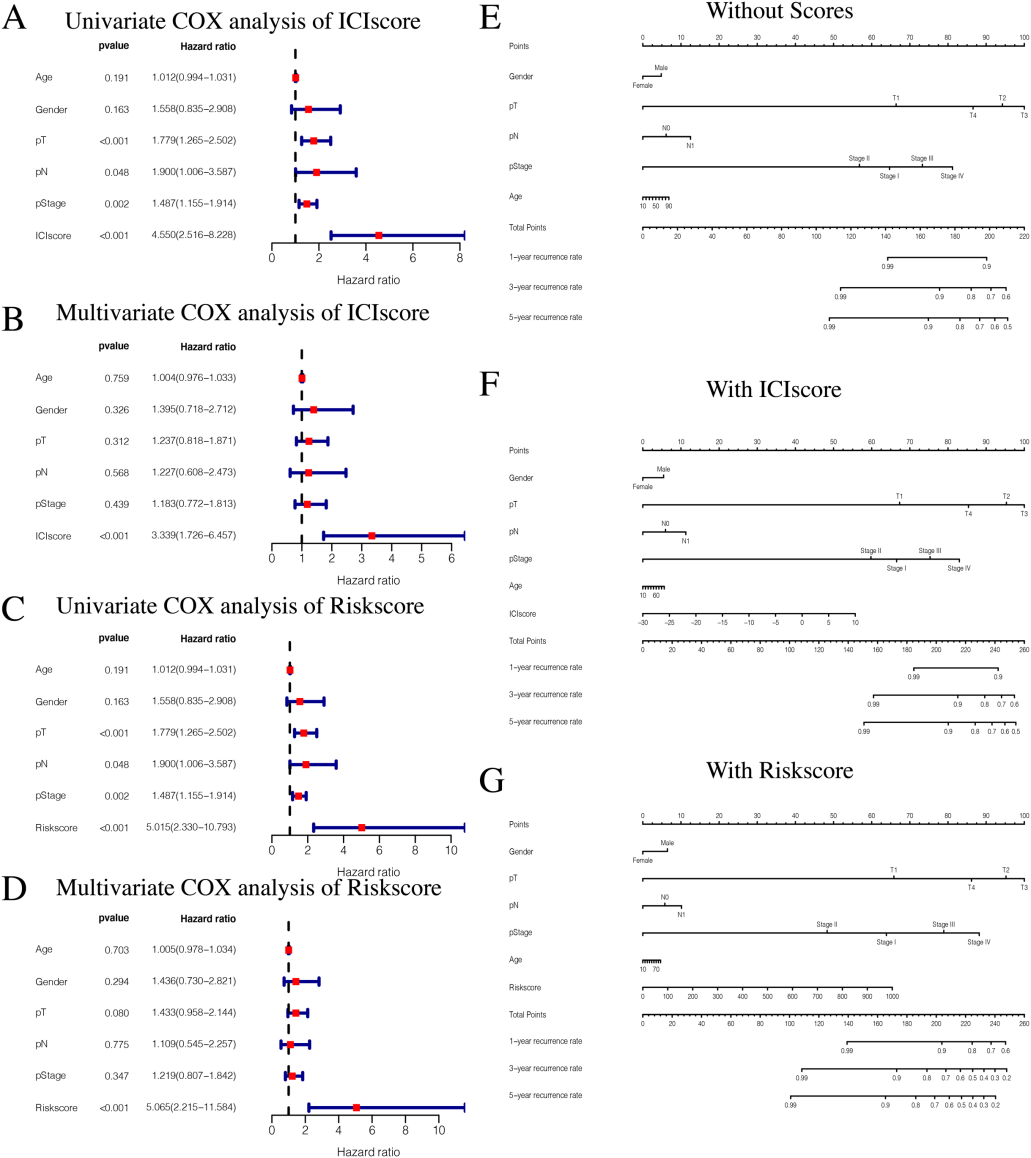
Independent prognostic analysis of ICIscore and Riskscore. (A) Univariate COX analysis of ICIscore about PTC prognostic signatures and clinical characteristics. (B) Multivariate COX analysis of ICIscore about PTC prognostic signatures and clinical characteristics. (C) Univariate COX analysis of Riskscore about PTC prognostic signatures and clinical characteristics. (D) Multivariate COX analysis of Riskscore about PTC prognostic signatures and clinical characteristics. (E) The nomogram of different clinical traits of the patients without scores. (F) The nomogram of different clinical traits of the patients with ICIscore. (G) The nomogram of different clinical traits of the patients with Riskscore.

While exploring potential independent prognostic factors, we did not include pM stages in this study due to the presence of many missing values in pM stages. Using univariate and multivariate COX analysis, we found that ICIscores and Riskscores could be used as independent prognostic factors (Figs. 10A–10D). Additionally, pT, pN, and pStage may also be potential prognostic factors according to univariate COX analysis with *P* value < 0.05. The nomograms showed the different clinical traits of patients without scores,with ICIscore or with Riskscore (Figs. 10E–10G). This provides a clinical reference for these patients’ prognosis.

## Discussion

Immune cells play an important role in tumor prevention, initiation, and progression (Crespo et al., 2013). The majority of PTC patients have excellent prognoses, but there are patients who experience disease recurrence and progression. Therefore, it is necessary to analyze the prognosis of all PTC patients.

We first obtained clinical data from 481 PTC patients with effective DFS time from TCGA database. Using the ssGSEA algorithm, we obtained enrichment scores for the tumor-infiltrating immune cells in each PTC sample. The PTC patients were divided into three ICI subtypes using consistent clustering. The results showed that almost all items were positively correlated, and that the most significant correlation was observed between MDSCs and other cells, particularly between MDSCs and effector memory CD8T cells. MDSCs are a subset of functional myeloid cells with immunosuppressive properties. They are the precursor of dendritic cells (DCs) and macrophages and/or granulocytes, and they have the ability to significantly inhibit the immune cell response. MDSCs exert immunosuppressive functions through a variety of pathways and mechanisms, and can suppress lymphocytes by secreting Arg-1, iNOS, or ROS. Additionally, Treg can also be induced to indirectly inhibit the body’s immune response. MDSCs have been found in the peripheral blood of patients with several malignant and non-malignant diseases. They are produced during tumor progression and inhibit the antitumor function of T cells and natural killer cells (NK cells). Their abundance is associated with poor prognosis in cancer patients and poor immunotherapy outcomes (Weber et al., 2021). MDSCs have strong immunosuppressive potential and are an important component of the tumor microenvironment (TME). Tumor cells use MDSC’ inhibition mechanism to establish immunosuppressive TME, thereby inhibiting anti-tumor immune responses and promoting tumor progression (Kalathil & Thanavala, 2021). Our data further confirm the role of MDSCs as cancer surveillance tools. We compared the enrichment scores across the ICI subtypes, and the results showed that there were significant differences across the three ICI groups, indicating that our clustering would be valuable and instructive in clinical practice. Because ICIcluster 3 had the highest level of immune cell infiltration, we inferred that a high level of immune cell infiltration could be associated with the best prognosis. Notably, ICIcluster 2 showed moderate immune cell infiltration but was associated with the worst prognosis, while ICIcluster 1 had the lowest level of immune cell infiltration but was associated with a moderate prognosis. Considering that there may be potential molecular differences across different ICI subtypes, we screened out DEGs associated with the three ICI subtypes. Upregulated genes were defined as being highly expressed in at least two ICI subtypes with higher immune cells. We ultimately obtained 15 differentially expressed genes: 10 upregulated genes and five downregulated genes.

Through our consistent cluster analysis of the above 15 genes, we obtained three gene clusters. The immune cell infiltration levels showed that Genecluster 1 and Genecluster 3 had more tumor-infiltrating immune cells, and Genecluster 3 was more significant than Genecluster 1.

Additionally, prognostic differences across the three gene clusters were explored. Genecluster 1 and Genecluster 2 showed better prognoses, while Genecluster 3 was associated with the worst prognosis. However, in combination with previous results, Genecluster 3 had more immune-infiltrating cells, but had the worst prognosis. The role of the immune system in the development of cancer is worth further study, as its activation may have both positive and negative consequences. Both high and low immunity can promote tumor occurrence and development. The association between high immune levels and tumors may be related to an increase in immune escape. Researchers have studied the immune system’s reactivation when fighting cancer since the end of the 19th century. Cooley was the first to demonstrate that bacterial infections induced in cancer patients could eliminate, or at least slow down, the disease (Zhao et al., 2020). After several decades, the seminal work of the two 2018 Nobel Prize in Medicine winners, James Patrick Allison and Tasuku Honjo, further validated the use of immune checkpoint inhibitors in cancer treatment (Coley, 1991). Because of these findings, many immune checkpoint inhibitors have received FDA approval for cancer treatment. It could be said that suppressing the level of immune cell infiltration to treat cancer is now a universally accepted fact. However, some studies have shown that low levels of immune cell infiltration are associated with a better cancer prognosis (Aaes & Vandenabeele, 2020). This opposing conclusion suggests that the immune system has two functions during tumor development. Therefore, it is necessary to further study the effect of immune cell infiltration levels on tumors.

According to the results described above, the ICIscore and Riskscore immune infiltration levels were highly consistent, suggesting similarities across most of the pathways enriched by GSEA. This was also shown in previous research by Subramanian et al. (2005).

We calculated ICIscores for each PTC sample and used the optimal ICIscore cutoff value in order to divide the patients into two groups (high ICIscore and low ICIscore). The results showed that patients with high ICIscores were mainly distributed in Genecluster 3 with poor predicted prognoses. In order to verify whether ICIscores were related to differences in immune levels between the two groups, we compared the tumor-infiltrated immune cells between the two groups using the Mann–Whitney *U* test. We also selected some immune checkpoint-related genes and immunoactive factors. The Mann–Whitney *U* test showed that, except for PDCD1 and TNF, most of the immune checkpoint-related genes and immune active factors were highly expressed in the ICIscore high group (Leach, Krummel & Allison, 1996). This again demonstrated the association between high immunity and poor prognosis.

To further explore the potential prognostic value of the genes, we constructed the prognostic characteristics of five mRNA. Studies have shown that MMP9 is associated with KDM1A and Inava. KDM1A can apparently inhibit the expression of TIMP1 by demethylating H3K4ME2 in the TIMP1 promoter region, activating MMP9, which is responsible for tumor migration and PTC invasion through this pathway (Zhang et al., 2019). The expression levels of AKR1C1 family members is strongly correlated with the malignant transformation of tumors and drug resistance to tumor therapy (Zeng et al., 2017). PLA2G2E is mainly related with obesity. It changes small amounts of lipoprotein phospholipids, phosphatidylserine, and phosphatidylethanolamine to moderately promote lipid accumulation in liver and adipose tissue (Sato et al., 2014). CARTPT is correlated with the neuroendocrine function of the hypothalamus, and may affect TSH levels (Burgos, Iresjö & Smedh, 2016). SLC5A1 plays a role in transmembrane glucose transport and encodes SGLT1. Abnormal SLC5A1 expression has been observed in many different types of cancer (Gao et al., 2019; Mojica, Luna-Vital & Gonzalez de Mejia, 2018; Lei et al., 2016).

We specified the median Riskscore value of the training set as the cut off and fit in the testing set and total set. There were significant differences in the infiltration levels of immune cells between the two groups, and the group with high Riskscore showed more immune cell infiltration. Additionally, most of the checkpoint-related genes and immune activity factors, except IL1A and IL6, were highly expressed in the Riskscore high group. This suggests that immune system activation may be both harmful and beneficial to cancer prognosis, which is consistent with previous research (Leach, Krummel & Allison, 1996).

We constructed ICIscore and Riskscore signatures using a PCA algorithm and COX-PH algorithm, respectively. However, the advantages and disadvantages of these two algorithms are still worth exploring and comparing.

When combined with the above two models, the ICIscore *p*-value was smaller, suggesting that ICIscores may be more accurate in predicting the prognosis of PTC patients, and Riskscores may be more suitable for most PTC patients. We concluded that combining these two scoring mechanisms could effectively provide patients with more accurate prognostic analyses. Our results show a direction for individualized clinical treatment of PTC patients in the future.Nevertheless, there are still limitations to our study. Since the TCGA database was the only databases that includes disease-free survival and recurrence time of PTC, we could not verify the results externally.Therefore, the results of our study need to be supported by a large number of clinical data.

## Conclusion

The results of this study showed that by combining the two constructed models, ICIscore and Riskscore, we made more accurate and reasonable inferences about the prognosis of PTC patients. This could potentially guide more effective and reasonable treatment plans for clinical PTC patients.

## Supplemental Information

10.7717/peerj.11494/supp-1 Supplemental Information 1 Clustering analysis. (a) Relative change in area under CDF curve in ICIcluster from TCGA sample. (b) Consensus matrix CDFs from k = 2 to 8 in ICIcluster from TCGA sample. (c) Consensus matrixes for k = 3 in ICIcluster from TCGA sample. (d) Relative change in area under CDF curve in Genecluster from TCGA sample. (e) Consensus matrix CDFs from k = 2 to 8 in Genecluster from TCGA sample. (f) Consensus matrixes for k = 3 in Genecluster from TCGA sample. (g) Relative change in area under CDF curve in ICIcluster from GEO combine set. (h) Consensus matrix CDFs from k = 2 to 8 in ICIcluster from GEO combine set. (i) Consensus matrixes for k = 3 in ICIcluster from GEO combine set.

10.7717/peerj.11494/supp-2 Supplemental Information 2 Exploration and validation of the correlation between differentiation of ICI subtypes and immune cell infiltration in GEO combined set. Through ssGSEA,28 immune-infiltrating cells were enriched.(a)The heat map is included the sample (GSE33630, GSE35570, GSE60542), tumor purity, estimate score, immune score, stromal score and ICIcluster. (b) The correlation matrice of tumor-infiltrating immune cells. (c) The comparation of enrichment score among three ICI subtypes. (d) The expression level of PD-L1 in each combination was compared. (e) The expression level of CTLA4 in each combination was compared.

10.7717/peerj.11494/supp-3 Supplemental Information 3 Prognostic correlation analysis of three ICI subtypes in GEO combined set. (a, b) Enrichment plots showing different enrichment of different diseases and pathways in the Rank in Ordered Dataset.

10.7717/peerj.11494/supp-4 Supplemental Information 4 Differential gene analysis. The up-regulated and down-regulated expression levels of differentially expressed genes in TCGA set

10.7717/peerj.11494/supp-5 Supplemental Information 5 Tables S1–S7.

## Abbreviations

PTC: Papillary thyroid carcinoma
ICI: Immune cell infiltration
DFS: disease-free survival
ssGSEA: Single Sample Gene Set Enrichment Analysis
CTLA-4: Cytotoxic T-lymphocyte antigen 4
PD-L1: Programmed cell death-Ligand 1
LogFC: Log2 fold change
FDR: false discovery
PCA: principal-component analysis
K-M curve: Kaplan-Meier curve
ROC: receiver operating characteristic curves
GO: Gene Ontology
KEGG: Kyoto Encyclopedia of Genes and Genomes

## Additional Information and Declarations

### Data Availability

The following information was supplied regarding data availability: The raw data are available at TCGA (TCGA-THCA) and GenBank: GSE33630, GSE35570, GSE60542.
