# Supplementary figures and images for: The landscape of tumors-infiltrate immune cells in papillary thyroid carcinoma and its prognostic value
Source: PeerJ. 2021 May 21;9:e11494. doi: 10.7717/peerj.11494 (PMC8142931; doi:10.7717/peerj.11494)

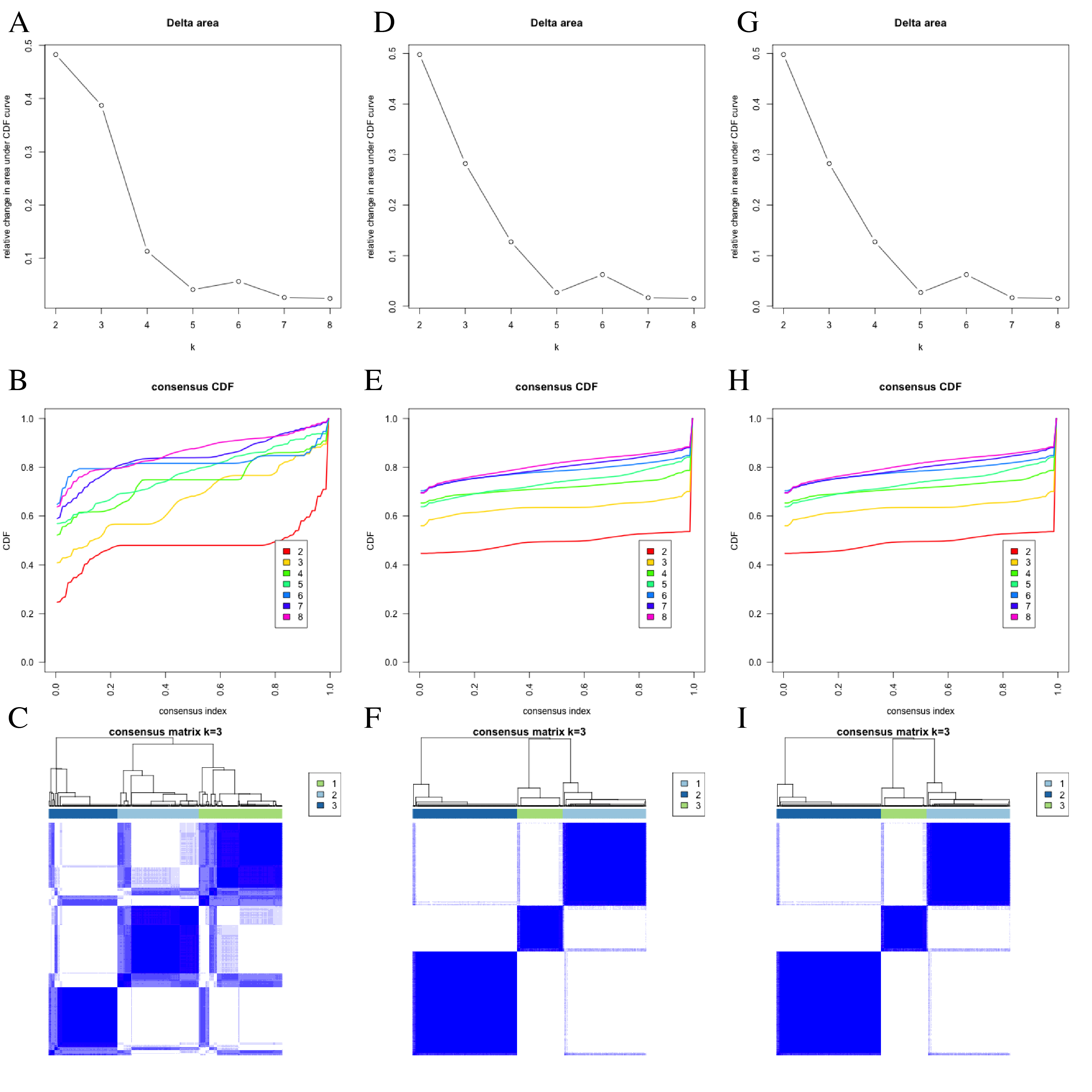

Supplement: Supplemental Information 1 — (a) Relative change in area under CDF curve in ICIcluster from TCGA sample. (b) Consensus matrix CDFs from k = 2 to 8 in ICIcluster from TCGA sample. (c) Consensus matrixes for k = 3 in ICIcluster from TCGA sample. (d) Relative change in area under CDF curve in Genecluster from TCGA sample. (e) Consensus matrix CDFs from k = 2 to 8 in Genecluster from TCGA sample. (f) Consensus matrixes for k = 3 in Genecluster from TCGA sample. (g) Relative change in area under CDF curve in ICIcluster from GEO combine set. (h) Consensus matrix CDFs from k = 2 to 8 in ICIcluster from GEO combine set. (i) Consensus matrixes for k = 3 in ICIcluster from GEO combine set. [file peerj-09-11494-s001.png]

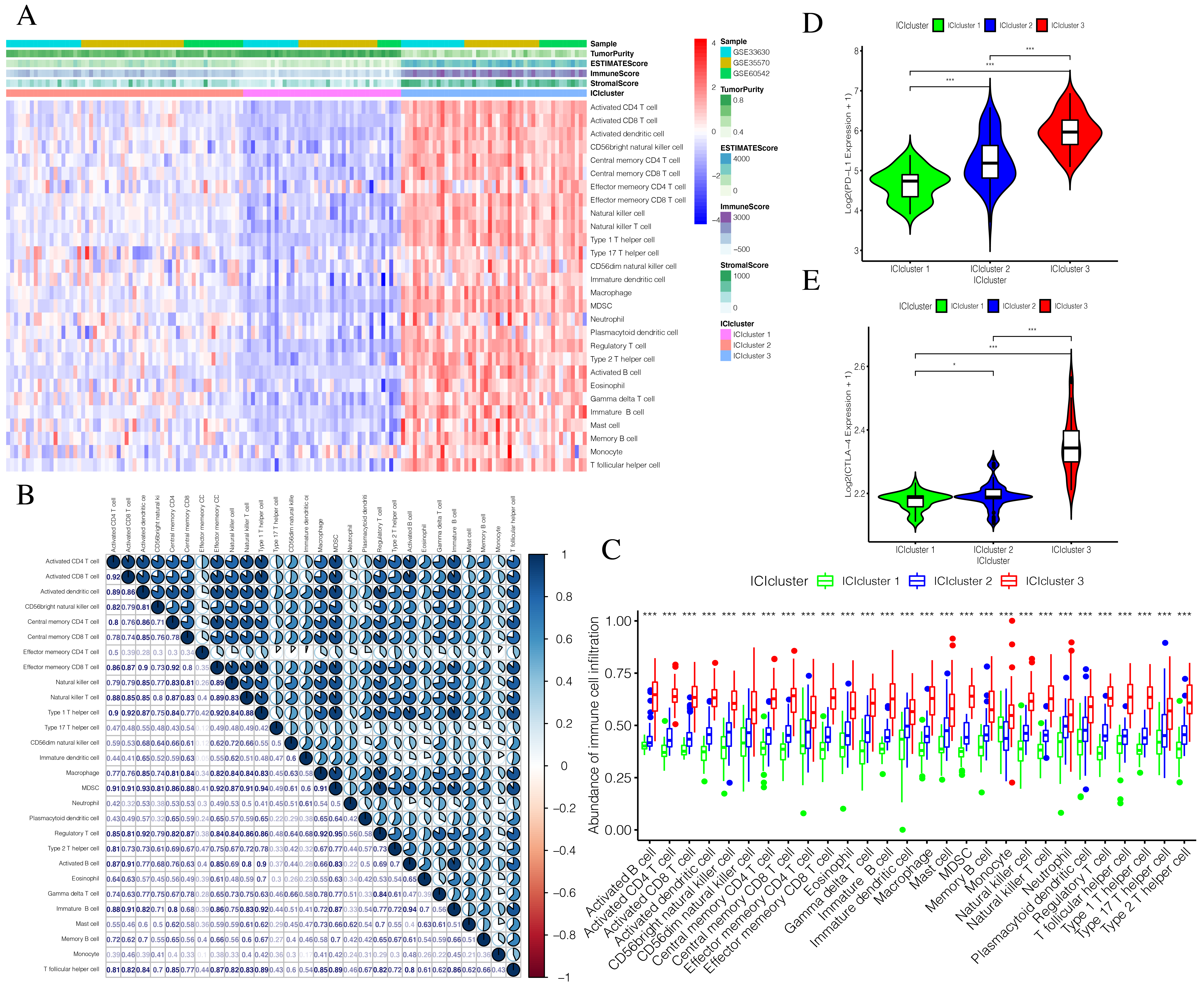

Supplement: Supplemental Information 2 — Through ssGSEA,28 immune-infiltrating cells were enriched.(a)The heat map is included the sample (GSE33630, GSE35570, GSE60542), tumor purity, estimate score, immune score, stromal score and ICIcluster. (b) The correlation matrice of tumor-infiltrating immune cells. (c) The comparation of enrichment score among three ICI subtypes. (d) The expression level of PD-L1 in each combination was compared. (e) The expression level of CTLA4 in each combination was compared. [file peerj-09-11494-s002.png]

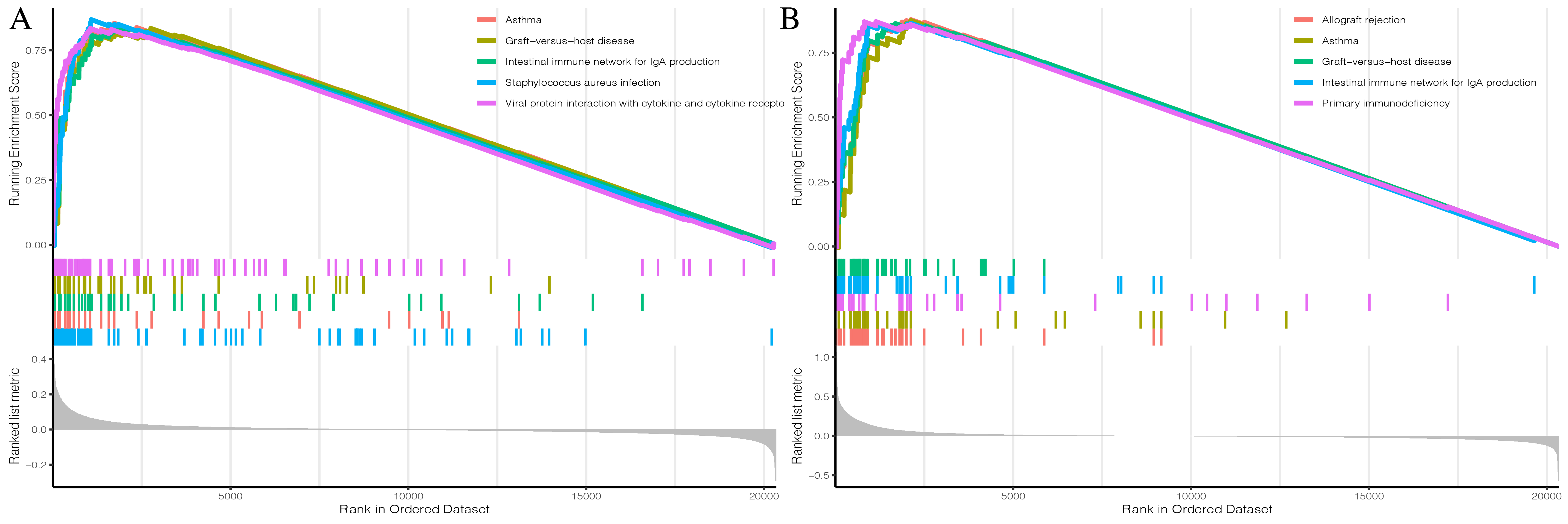

Supplement: Supplemental Information 3 — (a, b) Enrichment plots showing different enrichment of different diseases and pathways in the Rank in Ordered Dataset. [file peerj-09-11494-s003.png]

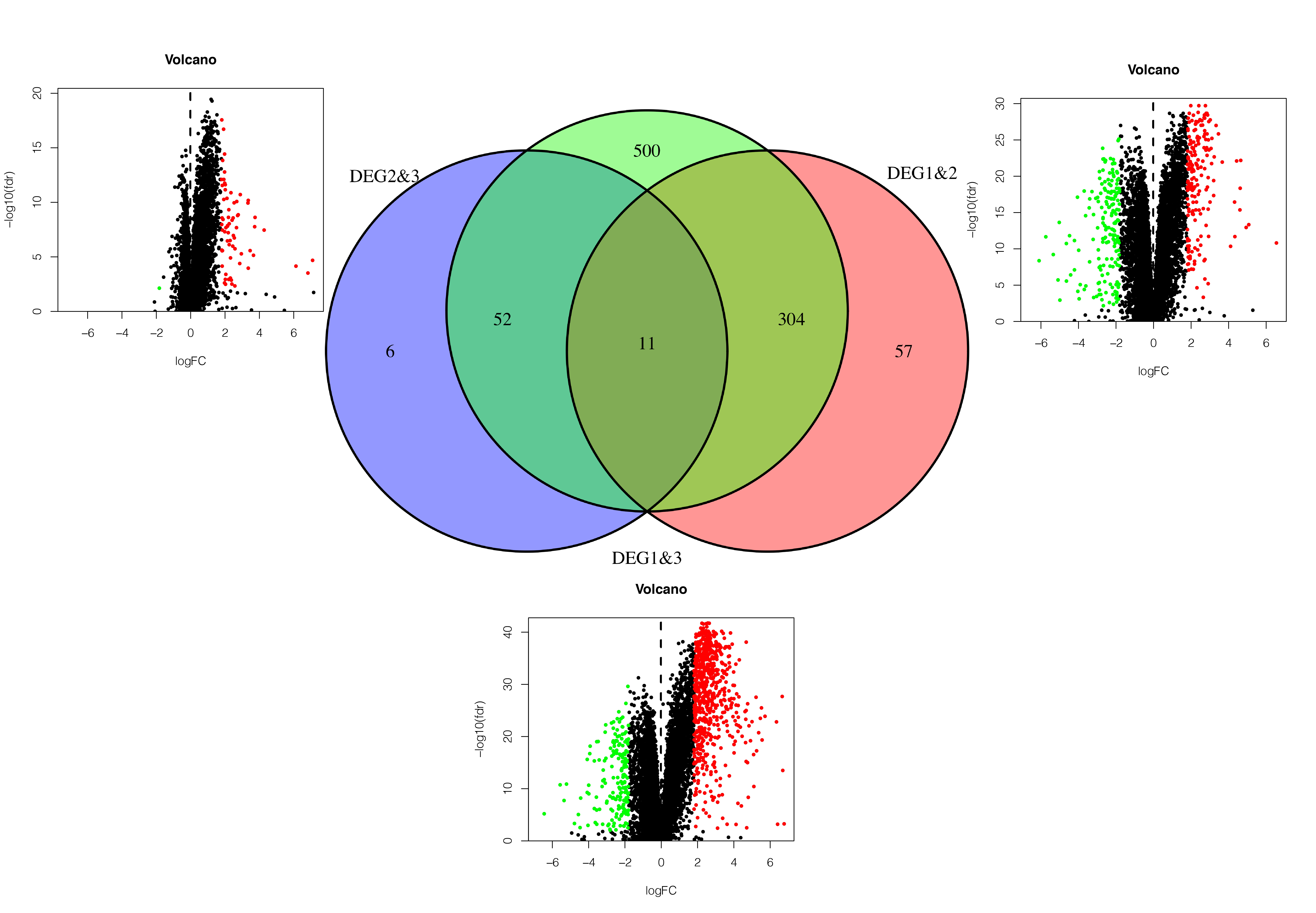

Supplement: Supplemental Information 4 — The up-regulated and down-regulated expression levels of differentially expressed genes in TCGA set [file peerj-09-11494-s004.png]
